# Attunement and Paternal Characteristics in Care Relationships in the Presence of Children Diagnosed with Autism

**DOI:** 10.3390/ijerph18042010

**Published:** 2021-02-19

**Authors:** Magda Di Renzo, Viviana Guerriero, Andrea Pagnacco, Massimiliano Petrillo, Lidia Racinaro, Simona D’Errico, Federico Bianchi di Castelbianco

**Affiliations:** Institute of Orthophonology (IdO) of Rome, 00198 Rome, Italy; viviana.guerriero@gmail.com (V.G.); andrea.pagnacco@gmail.com (A.P.); m.petrillo@ortofonologia.it (M.P.); l.racinaro@ortofonologia.it (L.R.); s.derrico@ortofonologia.it (S.D.); f.bianchi@ortofonologia.it (F.B.d.C.)

**Keywords:** autism spectrum disorder, fathers, parental attunement, caring abilities

## Abstract

Parents of children with autism spectrum disorder (ASD) have to address various challenges mainly due to their children’s atypia related to communication, emotion regulation and behaviors, arising also within the relationship with their caregivers. Several studies have pointed out that children with ASD can exhibit many difficulties regarding initiating and maintaining meaningful relationships with others. To date, little research has explored the interactions between children with ASD and their fathers, focusing more on mothers. In the context of parent–child interactions in the presence of autism, recent studies have highlighted the importance of parental attunement but there is little research considering solely the affective-bodily dimension. Therefore, the aim of the present study was to explore the parental attunement in fathers of children with ASD observed during play interactions and to investigate the relationship between paternal attunement and the perception of their psychological characteristics related to care relationships. The results highlight that fathers who describe themselves as better in affective care and sensitivity toward others more likely have an absence of paternal attunement during play interactions. The data presented are discussed in the light of intervention hypotheses, aimed at improving the relationship between fathers and children with autism.

## 1. Introduction

Autism spectrum disorder (ASD) represents a heterogeneous neurodevelopmental set of conditions with onset in early childhood characterized by persistent deficits in communication and social interaction and the presence of restricted and stereotyped behaviors, interests or activities [1,2]. Such symptom patterns in children with ASD are extremely variable leading to many difficult challenges for parents [3,4,5]. The difficulties of these children, mainly due to atypia related to (social) communication, responses to stimuli and regulation of emotions [6], can have a significant impact on initiating and maintaining meaningful relationships with others including with their parents [7]. However, it should be noted that most of the research that has deepened the study of the interactions between children with ASD and parents is mainly based on investigation of the mother–child relationship [8,9,10,11,12,13], leaving the father–child relationship more in the background. Over the last decade, attention of the scientific community on the specific role and psychological functioning of fathers during interactions with their children has increased, even in the presence of neurodevelopmental disorders such as in the case of ASD [14,15], as well as their influence on the outcomes of therapeutic interventions targeting these children [16].

Although there is still little research relating to the involvement and intervention of fathers, the data available would seem to highlight that fathers can be as effective as mothers in implementing interventions aimed at their children during their interactions with their children with ASD [16] and can represent a resource for the improvement of the communication, social and symbolic play skills as well as the emotional regulation of these children [14,15]. However, the literature deepening the parent–child relationship with ASD has highlighted in parents the feeling of being ineffective or incapable of raising their children [16]. Fathers in particular show great difficulty in accepting the diagnosis of their children [17,18,19,20] with high levels of anxiety, stress and anger [21,22,23], more directive and less active behaviors involving their children [16,24,25] and a reduced initiative in play situations [15]. These are all aspects that could negatively affect their ability to take care of their children [26,27,28,29].

Recent studies on parent–child interactions have also underlined the importance of parental attunement, which is understood to be the ability of parents to be sensitive to their child’s signals, to understand and respond to them appropriately and to adapt to their needs [19]. During parent–child interactions, attuned parents are able to simultaneously consider the motor, cognitive, social, emotional and communicative skills of their child [30], playing the role of promoting their child’s development in terms of their ability to self-regulate and their capacity to be engaged in relationships with others [19].

As children with ASD often show specific difficulties in processing experiences, responding to social interactions and using symbolic play as well as engaging in unusual behaviors and reactions, to be attuned with their children may represent a particular challenge for parents [3,31]. During infant–parent interactions, infants are able to form mental representations mainly based on emotional and somatic components, hereafter referred to as the affective-bodily dimension [32]. To better understand the interactions with their children with ASD, parents should mostly consider the affective-bodily dimension of individuals with ASD in different life situations [33]. However, few studies have investigated the bodily dimension as central to the attunement between the caregiver and the child.

Many years ago, Winnicott [34] underlined the importance of the body dimension by highlighting the “environmental provision that corresponds loosely with the establishment of a psycho-somatic partnership” (p. 62) or “handling” in the first dyadic interactions. Based on these early hypotheses and in the wake of the studies within infant research, Stern [35] emphasized the cross-modal exchange of early dyadic interactions; that it is a match between the partners using a different mode of expression focused on the internal state and not on the manifest behaviors. Caregiver and infant interactions are characterized on the ‘how’ (rather than the ‘what’); that is, a correspondence of the intensity and temporal or spatial modality present in the behavior of both participants. The parent, according to Stern, passes from one sensory modality to another by sharing the dynamic form of the relationship, faithfully preserving the duration, speed and time and maintaining a match between the “dynamic forms of vitality” (p. 17) [35]. The “dynamic forms of vitality” are perceptual motor gestalt conveying vital affects or the affective exchanges between mother and child. During infant–parent interactions, infants are able to form mental representations mainly based on emotional and somatic components or the affective-bodily dimension [32].

In cases of children with neurodevelopmental disorders including children with autism, recent research has shown that parental attunement is associated with more positive attitudes toward children’s independence and that a greater knowledge of children’s development is related to parental attunement [36]. In a study involving 45 preschool children with ASD, the maternal sensitivity during parent–child play mediated the association between maternal insightfulness/resolution of the diagnosis and the child’s attachment [37], suggesting the importance of parental abilities to positively support the mother–child relationship. Subsequent studies have shown that lower levels of emotional attunement and synchrony in parents during naturalistic play are associated with higher ASD symptomatology [38] and that mothers who accept their child’s diagnosis are more likely to be sensitive to their children while playing [39]. A 2018 study [32] was conducted with a sample of 11 fathers of children with ASD using a narrative methodology and the fathers’ play interactions with their children were investigated. The data showed that parents who were more attuned and capable of accepting their children’s play interests and who showed less adherence to the social norms of the play were also more accepting during the play narratives. The results of this study also showed that fathers used a directive/active approach during play and reshaped their expectations and ideas of play in response to the affective-bodily and social-spatial experiences of their children in their living environment. In our recent study [19], we found that those parents, both mothers and fathers, who were better able to accept their children’s diagnosis of ASD and to understand their point of view were also better able to become attuned during play interactions with their children.

In the light of the literature presented here, the aim of this research was to investigate the psychological characteristics of fathers in care relationships and their ability to become attuned with their children with ASD during play interactions, placing particular emphasis on the affective-bodily dimension.

## 2. Materials and Methods

### 2.1. Participants and Procedure

The sample consisted of 30 fathers of children receiving treatment at the Institute of Orthophonology (IdO) in Rome, Italy (Table 1). The fathers were, on average, 41.77 years of age (SD = 7.07) with 83.3% being married or cohabiting and 16.7% being separated/divorced. The children had an average age of 47.10 months (SD = 15.23) and 86.27% were male. At the time of participation in this study, the children had received a diagnosis of ASD and were undergoing therapy at the IdO within the DERBBI (Developmental, Emotional Regulation and Body-Based Intervention) intervention project [40] for an average of 14.30 months (SD = 14.64). The diagnoses of ASD were made by a multidisciplinary team with many years of experience using a global assessment that considers both the core and secondary aspects of autism [41]. The Autism Diagnostic Observation Schedule, Second Edition (ADOS-2) is a fundamental part of this assessment procedure. At the beginning of this study, the fathers provided informed consent for participation in this research project and received instructions about the procedure and the general objectives. The parents were also informed about the possible risks of participating in the study and the possibility of refusing to participate at any time, in accordance with the Declaration of Helsinki [42]. This study complied with the ethical guidelines and legal requirements of the country in which it was conducted. The research also adhered to the ethical standards of the American Psychiatric Association (APA). The study was approved by the Internal Ethics Commission of the IdO.

### 2.2. Instruments

The CUIDA questionnaire (Cuestionario para la Evaluación de Adoptantes, Cuidadores, Tutores y Mediadores, Eng. Tr. Questionnaire to evaluate people taking care of others, guardians and mediators [43], Italian adaptation [44]) is a self-report questionnaire composed of 189 items that measure the cognitive, affective and social aspects fundamental for relationships aimed at assisting other people. It consists of 14 primary scales: Altruism, Openness, Assertiveness, Self-Esteem, Problem-Solving, Empathy, Emotional Balance, Independence, Flexibility, Reflexivity, Socialization, Tolerance to Frustration, Capacity to Establish Emotional Ties and Grief Resolution Capacity. These scales make up three secondary factors:

1. Responsible care, whose low score refers to the tendency to establish relationships of assistance that are poorly reflective, flexible and resolute. These people are not constant in achieving their goals, do not carry out the commitments and activities that they start, are not very responsible, are not balanced and have difficulty making decisions independently.
2. Affective care, whose low score corresponds to people who have difficulty offering emotional support and coping with situations in which it is essential to give and receive affection. They are unsatisfied with themselves in care relationships, fail to understand and accept the feelings of others and may lose their temper when dealing with daily problems. They are also not very affectionate or friendly toward others.
3. Sensitivity toward others, whose low score refers to people who have difficulty perceiving the needs of others and are not sufficiently interested in other people. They do not feel personally involved in their needs and do not give much importance to their emotions and how they feel. These caregivers are not very sensitive or affectionate and they do not try hard enough to help or solve the problems of others.

The CUIDA scales also allow the calculation of an additional factor—aggressiveness—a high score in which refers to subjects who tend to express their needs in a marked way to assert and defend their rights but who fail to consider the needs and rights of others. They have a low tolerance to frustration, reflection and flexibility and a difficulty in recognizing, understanding and accepting the feelings, interests and needs of others.

Scores in these scales and factors can be converted into one of three categories: high, medium or low. The original version of the questionnaire presented a reliability between Cronbach’s alpha (α) = 0.56 and α = 0.86 for the scales. The Italian version involved 1020 subjects and showed good internal consistency only for a few scales with a reliability ranging from *α* = 0.41 to *α* = 0.80. The CUIDA also showed a questionable *α* = 0.62 for aggressiveness and acceptable-to-good Cronbach’s alphas for the secondary factors; *α* = 0.77 for affective care, *α* = 0.79 for responsible care and α = 0.80 for sensitivity toward others.

The Dyadic Attunement Observation Schedule (DAOS) [19] is an observational measure of parent–child interactions during play. It consists of eight scales that make up a final score of parental attunement: (1) Joint Attention, (2) Body, (3) Interaction, (4) Space Sharing, (5) Play Sharing, (6) Autonomy, (7) Emotional Regulation, (8) Understanding a Child’s Mental States. Each scale has a score ranging from 0 to 3 (from absent to high). Attuned parents (score 3) can play with their children with sufficient participation (and absence of intrusiveness) with joint and alternating use of space (staying close to/far, next to/face-to-face, understanding the needs of the child and their sensory regulation) and an interactive exchange in which both can alternate in an active role, involving each other in the play. During the play, in fact, these parents activate a bodily dialogue made up of gestures, sounds and looks and are also able to facilitate their children’s actions without overlapping them with the aim of increasing their autonomy and supporting their abilities as well as allowing them to experiment with new actions. Attuned parents can recognize and repair moments of failed attunement and are able to act as a regulator of emotions for their child when they are unable to regulate them by themselves. Unattuned parents (score 0) on the contrary are not very proactive in involving the child in the game, they do not show a joint or alternating use of space (being close to/far and next to/face-to-face) and they are unable to activate a bodily dialogue (they appear awkward, sometimes blocked), tending to overlap the child or withdraw following requests for play. These parents are unable to repair moments of failed attunement, they may be irritated or frightened and are unable to contain or regulate their child’s emotions during the entire interaction or during moments of difficulty or tension.

This tool has been used with children with ASD, learning or language problems, emotional regulation disorders and with typically developing children. It is currently being validated. 

The Autism Diagnostic Observation Schedule, Second Edition (ADOS-2) [45,46,47] is a semi-structured, standardized assessment of communication, social interaction, play and restricted and repetitive behaviors for children aged from 12 months to adulthood. It presents various activities that elicit behaviors directly related to ASD and allows their assessment. The ADOS-2 takes into consideration two fundamental clinical areas; i.e., social affection (SA) and restricted and repetitive behaviors (RRBs), from the sum of which a total score is obtained. The comparison score (CS), on the contrary, allows a standardized assessment of the evolution of autistic symptoms over time for children of different ages and abilities. The ADOS-2 has been shown to have good psychometric properties, demonstrating its reliability in distinguishing individuals with ASD from those of other clinical groups [48,49,50,51].

### 2.3. Data Analysis

The authors used Spearman’s correlations to check the relationship between the children’s age, the fathers’ age, the duration of treatment, the ADOS-2 scores and the variables of our study (factors and scales of the CUIDA and DAOS attunement). The effect size was interpreted in line with Cohen’s [52] text (small effect: 0.10 ≤ *r* < 0.30; medium effect: 0.30 ≤ *r* < 0.50; large effect: *r* ≥ 0.50). The Mann–Whitney U-test was used to check the association between the gender of the children with the variables of our study. For the Mann–Whitney effect sizes, we performed eta-squared (*η*^2^) interpreted according to Cohen’s guidelines [52].

A 3 × 4 cross-tabulation was created to examine the associations between the CUIDA factors and scales and the DAOS attunement final score. Here, again, we used the likelihood ratio (LR) when our data did not satisfy the hypothesis of having at least 80% of the cells with an expected count greater than five for the chi-squared tests. Statistical analyses were performed using SPSS version 24.0 for Windows (IBM Corp., Armonk, NY, USA).

## 3. Results

### 3.1. Descriptive Statistics

Table 2 shows the percentage distribution of the CUIDA factors and scales. For the factors, the percentages of fathers who fell into the low category were 10% for responsible care, 16.7% for affective care, 30% for sensitivity toward others and 46.7% for aggressiveness (additional factor). 

Furthermore, the scales in which less than (or equal to) 10% of the fathers who fell into the low category were Altruism, Self-Esteem, Emotional Balance, Independence, Reflexivity, Tolerance to Frustration and Grief Resolution Capacity. The scales in which less than 10% and less than (or equal to) 20% of the fathers who fell into the low category were Openness, Assertiveness, Problem-Solving, Reflexibility and Capacity to Establish Affective Ties. Finally, the scales in which more than 20% of the fathers who fell into the low category were Empathy and Sociability.

Table 3 shows the percentage distribution for the total attunement and all DAOS scales, showing that the absent category was reached by a higher percentage of fathers in the attunement scale; 80% of fathers were classified as absent, 16.7% as low and 3.3% as medium. The scales in which more than (or equal to) 50% of the fathers who fell into the absent category were Ability to Understand the Child’s Mental States, Body, Autonomy, Emotion Regulation and Space Sharing. Furthermore, the scales in which less than 50% of the fathers who fell in the absent category were Play sharing, Joint Attention and Interaction. None of the fathers fell into the high category.

### 3.2. Correlations/Associations between the Control Variables with DAOS Attunement and the CUIDA Factors and Scales 

Spearman’s correlations were performed to verify the relationship between the control variables (age of children, age of parents, length of treatment and ADOS-2 scores) with the attunement final score of the DAOS and the factors and scales of the CUIDA. 

The data analysis showed no correlation between the father’s age and the CUIDA factors, nor between the secondary or additional ones (Table 4). There was a positive and significant correlation with the CUIDA Emotional Balance scale such that the fathers’ Emotional Balance increased as their age increased (*r_s_* = 0.37, *p* < 0.05, medium effect size).

The children’s age displayed no correlation with the CUIDA factors but there was a positive and significant correlation with the CUIDA Tolerance to Frustration scale, such that paternal tolerance to frustration increased as the age of the child increased (*r_s_* = 0.37, *p* < 0.05, medium effect size). Moreover, a negative and significant correlation was found between the children’s age and the DAOS attunement scale (*r_s_* = −0.48, *p* < 0.01, medium effect size), such that the fathers’ attunement decreased as the age of the child increased.

No statistically significant correlations emerged between the length of treatment and the CUIDA scales and factors or DAOS attunement nor between the ADOS-2 scores and the CUIDA scales and factors or DAOS attunement. 

The Mann–Whitney U-test was performed to check the association between the gender of the children with the DAOS attunement and the CUIDA factors and scales. Statistically significant relationships emerged between the Reflexivity scale of the CUIDA and the gender of the children (U = 22.00, *p* < 0.05, *η*^2^ = 0.10) and the Self-Esteem scale of the CUIDA and the gender of the children (*U* = 24.00, *p* < 0.05, *η*^2^ = 0.10), with a higher chance that girls had fathers with high Reflexivity and Self-Esteem at CUIDA than boys but with a small effect size (Table 4). No statistically significant associations emerged between the gender of the children and the other CUIDA scales and factors or DAOS attunement. 

### 3.3. Associations between DAOS Attunement and the CUIDA Factors and Scales

We verified the associations between DAOS attunement and the CUIDA factors and scales. In Table 5, only the statistically significant results are reported. A statistically significant association emerged between the attunement final scale of the DAOS and the affective care factor of the CUIDA (LR = 12.18, *df* = 4, *p* < 0.05), for which fathers who obtained medium and high affective care scores were more likely to have absent parental attunement. In particular, the two scales that were found to have a significant association with paternal attunement and to contribute to the formation of this factor were Emotional Balance and Grief Resolution Capacity. Emotional Balance was statistically significant (LR = 9.83, *df* = 4, *p* < 0.05) so fathers with medium and high levels of Emotional Balance were more likely to have absent attunement. Furthermore, the association between DAOS attunement and the CUIDA Grief Resolution Capacity scale was statistically significant (LR = 12.06, *df* = 4, *p* < 0.05), for which fathers with a medium and high ability to overcome pain were more likely to have absent attunement.

Furthermore, the other CUIDA secondary factor that emerged as statistically significantly associated with DAOS attunement was sensitivity toward others (LR = 12.85, *df* = 4, *p* < 0.05). Looking at Table 5, the higher percentage of fathers with absent attunement also had a high sensitivity toward others. The other associations between the factors and scales of the CUIDA and paternal attunement were not significant and are not reported in the table.

## 4. Discussion

The results obtained by the use of the CUIDA show that most of the fathers involved in this study described themselves as being endowed with flexible, resolute and reflective caring skills. They also seemed to perceive themselves as being able to engage in their care skills with emotional balance and autonomy as well as to offer emotional support even in situations of high stress.

Furthermore, the data presented highlighted that in our sample the fathers’ perception of their ability to be responsible when caring for their children could depend, in particular, on the parental perception of being independent in making their own decisions, assuming their own responsibilities without the need for the approval of others (Independence scale), being capable of acting in a thoughtful, organized and not impulsive way (Reflexivity scale) and being able to tolerate frustration or to accept that a desire, a project or an expectation was not satisfied (Tolerance to Frustration scale).

A smaller percentage of fathers obtained high scores in the sensitivity toward the other factor compared with the other two factors mentioned above, suggesting that, albeit within a general perception of adequate caring skills, they could be less prone to recognizing themselves as being supportive in terms of understanding the needs of others and being interested in other people, being sensitive and affectionate toward the individual they care for and being committed enough to help and solve the problems of others. Finally, almost half of these parents obtained a medium level of aggressiveness in care relationships, meaning medium levels of controlling impulses, intransigence, a lack of flexibility and an inability to tolerate frustration.

The results that emerged from the DAOS highlighted that in our sample the abilities to activate a bodily dialogue made up of gestures, sounds and looks (Body scale), to facilitate the child’s actions without overlapping him/her (Autonomy scale) and to understand the mental states of the child (Understanding the Child’s Mental States scale) seemed particularly deficient in most fathers. The result of a very high percentage (80%) of fathers lacking in their ability to become attuned is not in line with the results obtained in our previous study [19]. This paper stated that 48% fathers and mothers of children with ASD were lacking in parental attunement during play interactions and showed that parents who were more able to accept their child’s diagnosis and to be insightful were more likely to also become attuned with their children diagnosed with ASD [19].

The data presented also showed an increase in the fathers’ perception of being emotionally balanced with increasing age. This is in line with studies showing a better ability for emotional regulation with people who are getting older [53,54,55]. Fathers of older children also described themselves as more able to tolerate frustration arising from an unrealized desire or expectation than fathers of younger children. A possible explanation of these results may be that older children may exhibit behaviors that could be more easily understood and acceptable to these fathers who may feel to have a better knowledge of their children and to be more comfortable with their children’s idiosyncrasies. The additional data that emerged from our analyses highlighted that the fathers of girls seemed to be more likely to have high reflexivity and self-esteem than the fathers of boys. These data should be taken with extreme caution as a small effect size emerged. The small number of girls compared with boys may have influenced these results and should therefore be further investigated in future studies involving a greater number of girls with ASD.

Finally, the exploration of the relationship between parental attunement and fathers’ characteristics of caring deserves particular attention. In our sample, fathers who perceived themselves as more able to offer emotional support to others seemed to be more likely to be lacking in attunement during play interactions.

In the affective care factor, the aspects of the perception of being able to control one’s tension and behavior (Emotional Balance scale) and to process and overcome pain related to suffering or loss (Grief Resolution Capacity scale) were those that could have a major impact. It would therefore seem that the perception of fathers who reported with respect to these specific characteristics in the context of the care and assistance of other people did not correspond with what was observed in the context of play interaction between fathers and children with ASD. This seemed particularly relevant as it suggested that fathers could describe themselves as more able to provide emotional support, to control their emotional reactions following emotionally strong experiences and to cope with the pain and frustration that they experienced following significant pain but in interactions with their children with ASD, they seemed to show an important difficulty in understanding their children’s signals and responding appropriately in the here and now of the interaction.

This was also an apparently counterintuitive finding when compared with studies presented on parental attunement showing a positive relationship between paternal attunement and paternal attitudes and abilities [19,36,37]. This discrepancy could depend on the fact that in the above research the abilities investigated were not measured with self-report questionnaires as they were in the case of the present study where the CUIDA investigated a self-perception of the paternal ability to take care of others.

In our view, what parents perceive as a generally caring behavior may not work with a child with ASD because in the absence of adequate bodily communication, the parent may fail to interact with the child in that pre-cognitive, pre-reflexive and pre-verbal area in which the child finds themselves carrying out their actions [32,34]. To be attuned with an autistic child means, in fact, grasping the rhythm, intensity and strength of vital forms that are expressed but through stereotypes or the search for unusual sensory interests to which it is difficult to attribute meaning [35]. Even fathers describing themselves as good in their sensitivity toward others and with an adequate ability to offer emotional support may not be able to attribute intentionality to their children’s behavior; instead, they appear dysfunctional and to fail to respond to the affective state underlying their children’s manifested behaviors. To the best of our knowledge, there are not many other studies considering parental attunement as essentially characterized by the affective-bodily dimension. In our previous article [19], it was found that parents better able to accept the ASD diagnosis of their child and therefore able to accept the loss of the “healthy child” in favor of a more integrated vision of their child with autism and with better insightful abilities were also better able to become attuned to their child during play interactions. As mentioned above, the apparent contrast between these results and the results of this study could depend on the fact that the tool used in this study investigated self-perceptions through a self-report while in our previous study, we administered an interview assessing the ability of the parents to accept the diagnosis of their child that was coded by an expert clinician.

The analysis carried out also showed that the paternal perception of being more capable of perceiving the needs and requirements of others was related to the paternal ability of attunement but this relationship should be further investigated to better understand the direction of this relationship.

This study has several limitations: due to the low sample size, it was impossible to establish causal links between the variables considered, suggesting caution is needed when generalizing and interpreting the presented results. The small size of our sample compared with the number of analyses carried out could also lead to a greater risk of type one errors. Future studies should replicate these results in larger samples of fathers of children with ASD to confirm the findings of this study. These first results, however, highlight the importance of combining observational measures with self-report measures to enrich and articulate the study of the functioning of fathers in cases of a diagnosing ASD in their children. Another study limitation is that it was not possible to establish whether the identified relationships were specific characteristics of the father–child dyad with children with autism. To address this question, future studies should explore these findings, comparing fathers of children with ASD with a control group of fathers with children affected by other disabilities. Finally, by way of extension of this study of paternal attunement, future research should include the study of specific characteristics of the relational functioning of these children and their fathers’ mental health status.

## 5. Conclusions

Reflecting on the results presented, it is possible to speculate that in our sample the fathers’ perceptions of their own characteristics regarding taking care of others might not correspond to their actual ability to grasp their children’s emotional states and to become attuned to his/her needs. These difficulties emerged during play interactions, which is when children with ASD usually exhibit their atypia and their interference in communication and interaction with others. The parental difficulties in being attuned to their children may be related to a parental limitation in understanding their children’s unique way of processing experiences, behaving in social interactions and using their body to experience and regulate emotions [3,6,31].

Based on these considerations this study could provide useful information to improve the interactions between fathers and children with ASD through the involvement of fathers in early intervention programs with the aim of improving paternal attunement. A useful intervention for this purpose could include the use of a video feedback (VF) technique for supporting fathers in acquiring a greater awareness of their difficulties with respect to becoming attuned to their children during play interactions despite their perceptions of being good in caring for their child. The VF technique is already being used successfully with parents of children with ASD [56] and it could be implemented as part of a short dyadic psychotherapy involving the father and the child in a cycle of play sessions. Specifically, the therapist could help the father to focus his attention on the use of the body within the dyadic relationship and the affective exchanges associated with these interactions, thus modulating and expanding the meanings and patterns of the play interaction [57]. In other words, helping fathers to understand that even in the stereotypes and unusual sensory behaviors of their children there is a communicative intention whose understanding could allow the parent and child to experience a more attuned and loving relationship. 

## Figures and Tables

**Table 1:** Characteristics of the sample.

Characteristics of the Sample	*n* (%)	Mean (SD)
Gender, % Male	26 (86.7)	
Marital Status		
Married/Cohabitants	25 (83.3)	
Separated/Divorced	5 (16.7)	
Education Level of Fathers		
Compulsory School	4 (13.3)	
Professional Degrees	2 (6.7)	
High School Diploma	11 (36.3)	
Degree or Post-Graduate Degree	9 (30.0)	
Other or Not Responding	4 (13.3)	
Fathers’ Age, Years		41.67 (7.07)
Children’s Age, Months		47.10 (15.23)
Length of Treatment, Months		14.30 (14.64)
ADOS-2 Score		17.77 (6.79)

Note: ADOS-2 score = Autism Diagnostic Observation Schedule 2, total score.

**Table 2:** Percentage distribution of CUIDA factors and scales (Cuestionario para la Evaluación de Adoptantes, Cuidadores, Tutores y Mediadores, Eng. Tr. Questionnaire to evaluate people taking care of others, guardians and mediators [43], Italian adaptation [44]).

Percentage Distribution of CUIDA Factors and Scales	Low (%)	Medium (%)	High (%)
Secondary Factors			
Responsible Care	10.0	36.7	53.3
Affective Care	16.7	46.7	36.7
Sensitivity Toward the Other	30.0	40.0	30.0
Additional Factor			
Aggressiveness	46.7	40.0	13.3
Scales			
Altruism	10.0	63.3	26.7
Openness	20.0	56.7	233
Assertiveness	16.7	43.3	40.0
Self-Esteem	6.7	63.3	30.0
Problem-Solving	16.7	46.7	36.7
Empathy	26.7	63.3	10.0
Emotional Balance	10.0	50.0	40.0
Independence	10.0	43.3	46.7
Flexibility	13.3	53.3	33.3
Reflexivity	10.0	40.0	50.0
Sociability	30.0	40.0	30.0
Tolerance to Frustration	3.3	36.7	60.0
Capacity to Establish Affective Ties	16.7	46.7	36.7
Grief Resolution Capacity	6.7	56.7	36.7

**Table 3:** Percentage distribution of the Dyadic Attunement Observation Schedule (DAOS) scales.

Percentage Distribution of the Dyadic Attunement Observation Schedule (DAOS) Scales	Absent (%)	Low (%)	Medium (%)	High (%)
Attunement	80.0	16.7	3.3	-
Body	80.0	13.3	6.7	-
Interaction	26.7	73.3	-	-
Space Sharing	50.0	43.3	6.7	-
Play Sharing	36.7	56.7	6.7	-
Autonomy	56.7	36.7	6.7	-
Joint Attention	33.3	63.3	3.3	-
Emotional Regulation	50.0	50.0	-	-
Understanding the Child’s Mental States	93.3	6.7	-	-

**Table 4:** Correlations/associations between age of children, age of parents, length of treatment, ADOS-2 scores and the DAOS attunement and the CUIDA factors and scales.

Correlations/Associations between Age of Children, Age of Parents, Length of Treatment, ADOS-2 Scores and the DAOS Attunement and the CUIDA Factors and Scales	Fathers’ Age	Children’s Age	Length of Treatment	ADOS-2 Score	Children’s Gender		
	*r_s_*	*r_s_*	*r_s_*	*r_s_*	Boys (*n* = 26) Mean (SD)	Girls (*n* = 4) Mean (SD)	Mann– Whitney’s *U*
CUIDA Secondary Factors							
Responsible Care	0.22	0.26	0.19	−0.11	2.38 (0.70)	2.75 (0.50)	37.50
Affective Care	0.27	0.15	0.04	0.03	2.12 (0.71)	2.75 (0.50)	26.50
Sensitivity Toward the Other	0.19	0.12	0.07	−0.05	1.96 (0.72)	2.25 (0.96)	41.50
CUIDA Additional Factor							
Aggressiveness	−0.13	−0.27	−0.23	0.11	1.73 (0.57)	1.25 (0.50)	33.00
CUIDA Scales							
Altruism	0.25	0.16	0.18	−0.30	2.19 (0.66)	2.00 (0.82)	44.50
Openness	0.11	−0.12	−0.08	0.06	1.96 (0.75)	2.50 (0.58)	30.00
Assertiveness	0.24	0.19	0.09	−0.02	2.19 (0.54)	2.50 (0.58)	41.00
Self-Esteem	0.23	0.01	−0.05	−0.03	2.15 (0.71)	2.75 (0.50)	24.00 *
Problem-Solving	0.13	0.20	0.19	−0.11	2.12 (0.61)	2.75 (0.50)	26.50
Empathy	0.19	0.14	0.05	0.07	1.85 (0.65)	1.75 (0.50)	48.50
Emotional Balance	0.37 *	0.15	0.13	−0.08	2.23 (0.69)	2.75 (0.50)	29.50
Independence	0.08	0.19	0.06	−0.12	2.35 (0.67)	2.50 (0.58)	47.00
Flexibility	0.08	0.06	0.11	−0.09	2.15 (0.68)	2.50 (0.58)	38.00
Reflexivity	0.19	0.30	0.18	−0.08	2.30 (0.80)	3.00 (0.00)	22.00 *
Sociability	0.12	0.12	0.05	−0.05	1.92 (0.58)	2.50 (0.58)	31.00
Tolerance to Frustration	0.19	0.37 *	0.30	−0.09	2.54 (0.73)	2.75 (0.50)	42.50
Capacity to Establish Affective Ties	0.17	0.05	−0.10	0.10	2.15 (0.60)	2.50 (0.58)	39.00
Grief Resolution Capacity	0.20	0.15	0.02	0.22	2.27 (0.43)	2.50 (0.58)	42.00
DAOS Final							
Attunement	−0.15	−0.47 **	−0.25	0.17	0.23 (0.43)	0.00 (0.00)	40.00

Note: ADOS-2score = Autism Diagnostic Observation Schedule-2 scores, total score. * *p* < 0.05; ** *p* < 0.01.

**Table 5:** Significant associations between DAOS attunement and CUIDA factors and scales.

Significant Associations between DAOS Attunement and CUIDA Factors and Scales	DAOS Attunement				
	Absent (*n*, %)	Low (*n*, %)	Medium (*n*, %)	High (*n*, %)	LR
CUIDA Secondary Factors					
Affective care					
Low	4 (13.3%)	0 (0%)	1 (3.3%)	0 (0%)	12.18 *
Medium	9 (30.0%)	5 (16.7%)	0 (0%)	0 (0%)	
High	11 (36.7%)	0 (0%)	0 (0%)	0 (0%)	
Sensitivity Toward the Other					
Low	8 (26.7%)	0 (0%)	1 (3.3%)	0 (0%)	12.85 *
Medium	7 (23.3%)	5 (16.7%)	0 (0%)	0 (0%)	
High	9 (30.0%)	0 (0%)	0 (0%)	0 (0%)	
CUIDA Scales					
Emotional Balance					
Low	3 (10.0%)	0 (0%)	0 (0%)	0 (0%)	9.83 *
Medium	9 (30.0%)	5 (16.7%)	1 (3.3%)	0 (0%)	
High	12 (40.0%)	0 (0%)	0 (0%)	0 (0%)	
Grief Resolution Capacity					
Low	1 (3.3%)	0 (0%)	1 (3.3%)	0 (0%)	12.06 *
Medium	12 (40.0%)	5 (16.7%)	0 (0%)	0 (0%)	
High	11 (36.7%)	0 (0%)	0 (0%)	0 (0%)	

Note: LR = Likelihood Ratio. * *p* < 0.05.

## Data Availability

The data presented in this study are available on request from the corresponding author. The data are not publicly available due to their containing information that could compromise the privacy of research participants.
